# *Algorithmovigilance*, lessons from pharmacovigilance

**DOI:** 10.1038/s41746-024-01237-y

**Published:** 2024-10-02

**Authors:** Alan Balendran, Mehdi Benchoufi, Theodoros Evgeniou, Philippe Ravaud

**Affiliations:** 1 Université Paris Cité and Université Sorbonne Paris Nord, Inserm, INRAE, Center for Research in Epidemiology and StatisticS (CRESS), Paris, France; 2 INSEAD, Fontainebleau, France; 3 Centre d’Epidémiologie Clinique, AP-HP, Hôpital Hôtel-Dieu, Paris, France; 4 Columbia University Mailman School of Public Health, Department of Epidemiology, New York, NY USA

**Keywords:** Drug regulation, Standards, Machine learning

## Abstract

Artificial Intelligence (AI) systems are increasingly being deployed across various high-risk applications, especially in healthcare. Despite significant attention to evaluating these systems, post-deployment incidents are not uncommon, and effective mitigation strategies remain challenging. Drug safety has a well-established history of assessing, monitoring, understanding, and preventing adverse effects in real-world usage, known as pharmacovigilance. Drawing inspiration from pharmacovigilance methods, we discuss concepts that can be adapted for monitoring AI systems in healthcare. This discussion aims to improve responses to adverse effects and potential incidents and risks associated with AI deployment in healthcare but also beyond.

## Introduction

As artificial intelligence (AI) systems are increasingly used across applications and sectors, ranging from healthcare to energy, financial, transportation, and others, the focus is increasingly shifting to the post-deployment stages of the AI lifecycle to ensure the safe use of these systems^1^. Monitoring of AI systems is becoming increasingly critical^2^, and new tools and practices are being developed for this purpose, such as statistical tools or so-called AI incidents databases^3–5^. Indeed, the number of AI incidents is already large and is expected to increase. For example, 10.196 AI-related incidents and hazards have been identified since 2014^6^. While there is significant research on trustworthy AI^7^, there is only limited work on what happens *after* AI systems are deployed. Another limitation of AI monitoring is the lack of consideration of human or organizational factors^8^. For example, stakeholders like physicians who identify errors may lack effective feedback processes for reporting issues, hindering potential improvements to AI systems.

However, large-scale monitoring of technologies, broadly defined, is not a new challenge. Healthcare regulators, such as the US Food and Drug Administration (FDA), use processes and practices to monitor potential negative side effects of drugs in the market despite rigorous clinical trials in their pre-approval stages^9^. For instance, each year, regulators and manufacturers recall, on average, more than 1300 drugs, with more than half potentially causing temporary or serious health problems^10^. According to the FDA, a recall is defined as a manufacturer’s removal or correction of a marketed product that the FDA considers to be in violation of the laws it administers^11^.

Given the scale and potential of AI medical devices^12^, ensuring their proper monitoring and potential recall or withdrawal is necessary to limit their potential negative impact, similar to drugs. While AI is not the same as drugs, we argue that a lot can be learned from the field of pharmacovigilance^13^. We argue that drug development can also be seen as a kind of black box in some situations. For some widely prescribed drugs, it has been proven that their precise mechanisms of action sometimes remain unknown or not well understood, and, in some cases, have been secondarily proven to be different from what was initially thought^14^. Inspired by pharmacovigilance, which encompasses the assessment, monitoring, comprehension, and prevention of adverse effects associated with the use of drugs, Embi proposed in 2021, the term *algorithmovigilance* to transpose the same concepts for adverse effects associated with algorithms in healthcare^15^. Building on this term, we explore five key characteristics from pharmacovigilance that can be relevant for algorithmovigilance—also summarized in Table 1.

**Table 1:** Translating key concepts from pharmacovigilance to AI surveillance and monitoring (namely, *Algorithmovigilance*)

Practice	Pharmacovigilance	Algorithmovigilance
Post-approval evaluation	- Assessment done using observational data after post-market approval. ­- Investigates real-world safety in a larger and more representative population (larger population, more representative population, longer follow-up time, different dosages, and different outcomes) ­ - Assessment done at different points in a drug’s lifecycle (e.g. Periodic Update Safety Report (PSUR))	*What needs to be done:* - Assessment of model generalizability using external data after model approval - Identify model blind spots such as extreme/hard cases, racial bias, spurious correlations using data collected from different institutions, at different temporality, and for different tasks - Identify model impact on the clinical workflow (e.g. healthcare professionals and patients dissatisfaction, difficulty to use, perceived benefits)
Case reporting	*Who can report:* - Patients, caregivers, healthcare professionals, healthcare institutions, and drug manufacturers. *Content of report***:** - Patient information - Adverse reactions description - Information related to the suspected drug(s) - Information on management of the adverse reactions - Information about the reporter *Where to report:* - MedWatch - EudraVigilance	*What needs to be done:* - Case reports for adverse AI-system reaction (AAIR): - *Who can report*: - Patients, healthcare professionals, AI operators, AI researchers, hospitals, regulators, and AI system developers. - *Content of report*: - Patient information - Adverse reactions description - Information related to the AI systems (version details, hardware, software information) - Data preprocessing pipelines - Sequence of events leading up to the error and work environment - Details on other systems that impacted the model’s decision - Information on the AI developers - Information about the reporter - *Where to report:* - GitHub/GitLab for non-sensitive data - Private database for sensitive data
Data standardization	*ISO standard (HL7 27953-2:2011)* - Standardized framework for the reporting of case reports *Medical Dictionary for Regulatory Activities (MedDRA)* - Terminology developed for the evaluation of safety monitoring of medicinal products through all phases of the development cycle (i.e., from clinical trials to post-marketing surveillance). *Common Data Model* - Standardized vocabularies to harmonize the structure and content of observational data to enable efficient analyses and allow interoperability across various clinical domains.	*What needs to be done:* - Harmonization of data information (data acquisition devices and protocols, data quality control method, data annotation/labeling procedures, data management, population characteristics, and pipelines) - Harmonization of vocabulary used for model information (model specification, training information, and evaluation metric(s)) to allow interoperability across different hospitals
Causality assessment	*- Rechallenge/Dechallenge:* - Rechallenge (giving the drug again) or dechallenge (stopping the drug) to determine likelihood of causal relationship between drug and adverse event(s). - *Causality assessment:* - Classify potential relationships between drugs and suspected adverse drug reaction into different categories: - Certain, Probable/Likely, Possible, Unlikely, Conditional/Unclassified, Unassessable/Unclassifiable. - *Concomitant Drugs:* - Check for other concomitant drugs that may cause or be related to the adverse event(s)	*What needs to be done:* *- Reproducibility tests:* - Conduct reproducibility tests to identify the conditions under which the error occurred and to stress-test in alternative settings, identifying potential similar errors. - *Ablation studies:* - Perform ablation studies to pinpoint the component responsible for the incident (e.g., acquisition devices, preprocessing software, interpretability tools, biases in training data, human interpretation) - *Retraining/Redesigning*: - Consider potential retraining or redesigning of the AI system, incorporating new data or adjusting outcome variables in certain cases.
Adverse events dissemination	*For healthcare professionals:* *- Summary of Product Characteristics (SmPC)* accompanies authorized drugs, presenting key information on adverse events updated based on post-market authorization studies: - Adverse events are categorized based on seriousness and frequency: very common (≥1/10); common (≥1/100 to <1/10); uncommon (≥1/1000 to <1/100); rare (≥1/10,000 to <1/1000); very rare (<1/10,000). - Adverse events are stratified by specific groups of population (e.g. pediatric, elderly patients, patients with renal or hepatic impairment, and patients with other diseases or specific genotypes) - *For patients:* - *Patient Medication Information* and *Package leaflet* intended for patients in an understandable and concise way to ensure safe and effective use of drugs and prevention of side effects.	*What needs to be done:* *- Standardization of documentation for healthcare professionals and AI operators:* - Document that accompanies each AI-system in healthcare describing the different known adverse events in a tabulated format based on seriousness and frequency. - Indicate model risks and performance, or absence of reliable information, on specific patient subgroups (e.g. by age, race, gender, patients at high/low risks, patients with other comorbidities, etc.) - *Standardization of documentation for patients*: - Easily accessible and understandable document for patients informing them about the AI model’s functionality, the population on which the model has been tested, its impact on decision-making, and expected adverse events. - *Database to improve transparency:* - Database compiling the different known adverse events related to AI systems in healthcare. Incidents can be filtered by type of model, medical application, frequency, population at risk, and severity of the incident.

## Post-approval evaluation

Current AI monitoring techniques are mostly based on the use of statistical tests from data capturing the behavior (e.g., various error rates) of the AI systems. These methods typically detect potential shifts in the data/environment, such as covariate and concept shifts^16–18^. These tests are often automated (e.g., tests are computed every month or accuracy is aggregated over a couple of weeks long window), aligning with the emergence of MLOps aimed at operationalizing ML monitoring^3^. However, a significant number of FDA-authorized AI medical devices lack multi-site validation, and even fewer have undergone prospective evaluation^19^. This approach may inadequately reflect real-world performance—even within the same institution. Moreover, relying solely on algorithmic performance creates the risk of downplaying the importance of human factors who will be in “contact” with the AI system: patients, physicians, caregivers, AI operators, AI researchers, developers, hospitals, regulators, etc. For example, a study of an AI system for detecting diabetic retinopathy across multiple clinics in Thailand evaluated how the AI system affected patients and the way nurses made decisions based on the model’s predictions^20^. Current methods lack strategies to address post-deployment scenarios of AI systems.

For drugs, eventual studies conducted to evaluate the tolerance during the development of a drug are insufficient to identify all potential adverse reactions, especially rare adverse events, due to limited sample sizes (e.g., studies generally do not exceed 3000 participants) and non-representative trial population (e.g., does not include patients with more or other comorbidities, younger and older patients, use of concomitant drugs, etc.)^21^. In contrast, safety studies investigate drug safety in the real world across diverse conditions, including a larger population, more representative population, longer follow-up time, different dosages, and different outcomes^22^. For example, the Periodic Update Safety Report (PSUR) is a structured report that must be produced after its approval at different points in a drug’s lifecycle with updated information about its safety^23^.

Similarly, AI systems require evaluation beyond their pre-approval assessments. This should be carried out using diverse data from various sources, including data from the same institution but collected at different times (temporal generalizability), different institutions (geographic generalizability), and different clinical environments (domain generalizability)^24^. This is crucial to identify an AI system’s blind spots missed during pre-approval evaluation, such as hard or extreme cases, racial bias, or spurious correlations^25,26^. Human evaluation, assessing the AI system’s impact on the clinical workflow and patient (such as user dissatisfaction, difficulty in use, perceived benefits, etc.)^27–29^. should also complement pre-market evaluations. This process should ultimately result in a periodic update of the AI system findings based on the evidence learned from multiple validations, similar to the PSUR.

## Case reporting

When an adverse AI-system reaction (AAIR) occurs, it is important to report information related to the event. Current reports typically consist of automatic logs documenting each step of the AI system execution. However, these logs may lack context regarding the clinical environment in which the error occurred and prove challenging to interpret, hindering error reproduction.

For drugs, reporting of a suspected adverse event can be issued by anyone: patients, caregivers, healthcare professionals, and manufacturers. Reports can take different forms: spontaneous and voluntary reports, or mandatory reports. Each case report is populated not only by information about the drug and patient’s characteristics but also other information, such as the environment in which it occurred^8^. For a drug, this corresponds to information related to the suspected drug(s) (name of the drug(s), dosage, therapy date, batch number, reason for administration), description of the adverse reaction, information on the management of the adverse reactions (what assisted for the confirmation of the reaction, criteria for regarding the reaction as serious, use of other treatments after patient experiencing the adverse reaction, outcome of the adverse reaction), use of other concomitant drugs, the work environment, the personnel involved, information about the reporter, and any key contributing factors^30,31^. These reports are then collected, curated, and aggregated in publicly available databases like the FDA Adverse Event Reporting System (FAERS) through MedWatch or the EudraVigilance database from the EMA. Data protection is ensured by providing limited access based on the stakeholders^32^. These reports are critical as they are then used to make decisions about a drug, such as updating its labeling information or, in some cases, removing it from the market.

Similarly, reporting of incidents involving an AI system in healthcare should be accessible to anyone who will be “in contact” with the AI system: patients, healthcare professionals, AI operators, hospitals, AI researchers, regulators, and AI system developers. Methods to report AI-related suspected errors could be inspired by other mature communities. For instance, the software development community’s effective bug reporting, discussion, and tracking, facilitated by platforms like GitHub or GitLab, offer valuable insights. Sensitive data could be reported in private databases where privacy-preserving techniques such as differential privacy or synthetic data could be used to preserve data privacy while enabling robust reporting. Additionally, restricting access to case reports to designated entities responsible for analysis can enhance oversight and accountability. Reports should provide details on both technical errors and the clinical environment, ensuring comprehensive understanding and reproducibility of incidents. This may include specifics on the adverse event, version details of the AI system, hardware and software information, pipelines used to process the data, details on other systems that impacted the model’s decision (such as any acquisition devices, preprocessing tool(s), or interpretability tool(s)), latency, data quality assessment, difficulties or issues encountered when using the AI model, the sequence of events leading up to the error, the work environment, information on AI operators, and any other information.

## Data standardization

Data harmonization is essential in analyzing reports from various sources to reduce misclassification and information bias. For AI, the coding of data and concepts is based on the healthcare system adopted by the hospital or healthcare institutions. Discrepancies in coding standards can occur not only across different institutions but also within the same hospital when transitioning between different healthcare systems. Moreover, the standardization of AI development processes is also lacking. Risks of not having standardized concepts can hinder interoperability, particularly when multiple hospitals with different coding standards encounter the same AI system incident. The lack of uniformity in coding may result in disparate reports, hindering accurate incident assessment.

For drugs, to improve the analysis of drug-related adverse events, different strategies have been adopted to improve consistency across reports. For instance, the ISO/HL7 27953-2:2011 standard offers a common framework of data elements and a messaging format for transmitting adverse event reports. Additionally, initiatives like the Medical Dictionary for Regulatory Activities (MedDRA) propose common terminology for regulatory information exchange concerning medical products, including pharmaceuticals and drug–device combination products^33^. MedDRA has been translated into multiple languages to enhance interoperability across different linguistic contexts. Moreover, as discussed in the first section, evidence on drug safety can also be derived from observational studies, therefore, efforts to standardize terminology for observational data have been initiated. For instance, the Common Data Model project aims to standardize the structure and content of observational data, enabling subsequent analysis for drug safety surveillance^34^.

Similarly, coding information related to AI systems encompasses both data and model aspects. Information related to the data acquisition devices and protocols, data quality control method, data annotation/labeling procedures, data management, population characteristics, and pipelines should be consistent across different hospitals. Initiatives such as *Healthsheets* have started to identify the essential information that needs to be reported for a dataset in healthcare^35^. Moreover, standardizing information regarding the model development protocol, such as model specification, training details, and evaluation procedures (similar to Clinical Operations “ClinOps” for clinical trials) would enhance interoperability and facilitate comparisons among different AI systems.

## Causality assessment

Existing methods for monitoring AI systems are mainly focused on detecting deviations through statistical approaches. Less emphasis is placed on understanding and correcting the deviations. Errors related to AI systems can originate from various factors, including the data quality, the underlying bias in the data, acquisition devices, other software used in the workflow, medical treatments, and the skills and experience of the AI operators^16,25,26^. Undervaluing the importance of correcting AI system deviations may lead to situations where the system is disabled without attempting correction, adversely impacting the clinical workflow in which the AI system is integrated.

For drugs, investigating the relationship between a drug and a potential adverse effect is essential^36^. While reports of adverse events provide valuable safety signals, they only indicate a potential relationship between a drug and an adverse effect, which is why they are also often referred to as suspected adverse drug reactions. Investigating whether a drug actually caused an adverse event is important for several reasons: it can provide more evidence about the drug-adverse effect relationship, it can prevent a drug from being withdrawn if it is found to actually not be the cause of the adverse event, and it can allow the identification of other drugs that may provide a better explanation for the focal adverse event. Various methods, including clinical judgment and probabilistic methods, can be used to assess clinical plausibility^37,38^. For instance, one method used to determine the likelihood of a causal relationship between a drug and an adverse event is to conduct a rechallenge (or dechallenge) by giving again (or stopping) the drug to see if the adverse event reappears (or resolves). Reports are then classified into different causality categories: Certain, Probable/Likely, Possible, Unlikely, Conditional/Unclassified, Unassessable/Unclassifiable^39^.

Similarly, for an AI system, after an error has been detected, systematic reproducibility tests should be conducted to identify the exact conditions under which the error occurred and stress-test alternative settings, to identify potential similar errors. Ablation studies should be explored to identify the plausibility of each part of the AI system in the incident. In some cases, this could also motivate the retraining or redesigning of the AI system on a new batch of data or using a different outcome variable while ensuring (using the proper testing method) that the updated model does not result in a performance decline on existing data^16,25,40^. Versioning platforms such as GitHub and GitLab facilitate tracking model changes across versions. However, we note that current regulation mainly permits marketing approval for so-called locked AI systems^2^, although the FDA is investigating new regulations for adaptive AI systems in healthcare^41^. But this should not discourage the use of methods to control various aspects of a model after deployment, for instance, post-hoc explainability or fairness methods to investigate already deployed models without modifying or retraining them^25,26,42,43^.

## Adverse events dissemination

Effective dissemination of adverse events is crucial for preventing and mitigating their impact on end-users of AI systems. Several AI-related incident databases, such as the *Algorithmic and Automation Incidents and Controversies Repository (AIAAIC)* and the *AI Incident Database* have emerged^4,5^. However, these databases are not specifically tailored for incidents occurring in healthcare settings. Other efforts focus on presenting and summarizing key information about AI systems for decision-making^44,45^. For example, Sendak et al. introduced the “Model facts” labels, a one-page document summarizing key information about a machine learning model used in clinical settings^46^. Some challenges of those documents are that they primarily focus on model performance or are rarely intended for patients.

For drugs, access to comprehensive and easily readable information on adverse events is crucial to effectively prevent similar occurrences. In Europe, as part of the marketing authorization process, the *Summary of Product Characteristics* (SmPC) accompanies all authorized drugs, providing physicians with key information on safe and effective drug usage. In addition to general information such as the posology and the intended population, it also presents standardized, tabulated information on expected adverse events observed during clinical trials and after post-market authorization, using the MedDRA. Adverse events are categorized based on their seriousness and frequency: very common (≥1/10); common (≥1/100 to <1/10); uncommon (≥1/1000 to <1/100); rare (≥1/10,000 to <1/1000); very rare (<1/10,000). Moreover, adverse events are also indicated for specific subgroups (e.g., pediatric, elderly patients, patients with renal or hepatic impairment)^47^. Additionally, patient-oriented documents such as *patient medication information* and *package leaflets* offer concise information on safe drug usage and adverse effect prevention.

Similarly, dissemination of AAIRs could be done at different levels to effectively prevent future harm caused by AI systems. For example, a document could accompany each AI system in healthcare and include a description of the AI system, when and how to use it, general performance, and a tabulated summary of known adverse events that can be expected when using the AI system with the corresponding frequency and seriousness. The document could also include a section presenting the model performance on specific patient subgroups (e.g. by age, race, gender, patients at high/low risks, patients with other comorbidities, etc.). If the AI system has not been assessed for those subgroups, this should also be stated. These documents would benefit healthcare professionals and AI operators handling multiple systems. Moreover, easily accessible and understandable documents for patients could inform them about the AI system’s functionality, the population on which the system has been tested, its impact on decision-making, and expected adverse events. Both documents would be regularly updated with new validation on new data (see first section) and evidence of adverse events. Additionally, a public database could centralize information on known adverse effects, allowing users to filter healthcare-related incidents by type of AI system, medical application, type of error, frequency, seriousness, and population at risk.

## Differences between drug and AI development

While drug and AI system development shares similarities, we must also recognize their distinctions. First, the current state of AI development has led to a proliferation of models still lacking standardized validation, exacerbated by the absence today of tailored development or reporting guidelines—although this will likely improve over time^48^. This leaves the community with an excess of untested models. Another difference is that, unlike drugs, whose generalizability is mainly affected by variations in patients’ characteristics, the generalizability of AI systems depends on many more factors, including operational environments, clinical workflows, and integration with other IT systems. Updating AI systems is also inherently easier than updating drugs: AI systems can potentially be updated at varying frequencies and complexities, with different levels of performance guarantees^40^. Finally, unlike for drug safety studies, which use de-identified data to analyze data that minimizes concerns for privacy and confidentiality, the analysis of AI systems in healthcare requires access to patients’ EHR data, posing potential privacy challenges. Methods such as privacy-preserving machine learning, federated learning, or synthetic data generation can offer solutions for data analysis while preserving data privacy.

## Algorithmovigilance, the “pharmacovigilance” for AI

As increasingly more AI systems are being marketed in healthcare—and other industries—further attention may need to be shifted from the pre-marketing approval processes to the post-marketing risk management ones. Existing programs, such as MedWatch, may not be sufficient due to the evolutive nature of AI systems. As algorithmovigilance studies are emerging, lessons from pharmacovigilance may prove valuable and raise new research as well as products and practice directions^49^. While our focus is on AI systems in health, the ideas discussed herein are also applicable to the monitoring and surveillance of AI systems beyond healthcare.

### Reporting summary

Further information on research design is available in the Nature Research Reporting Summary linked to this article.

## Supplementary information

Reporting Summary
